# Data for simultaneous fermentation of galacturonic acid and five-carbon sugars by engineered *Saccharomyces cerevisiae*

**DOI:** 10.1016/j.dib.2020.105359

**Published:** 2020-03-02

**Authors:** Deokyeol Jeong, Suji Ye, Heeyoung Park, Soo Rin Kim

**Affiliations:** School of Food Science and Biotechnology, Kyungpook National University, Daegu, 41566, South Korea

**Keywords:** Citrus peel waste, Sugar beet pulp, Pectin, Metabolic engineering, CRISPR/Cas9, Bioethanol

## Abstract

*Saccharomyces cerevisiae* expressing heterologous pathways for xylose, arabinose, and galacturonic acid metabolism has been constructed by a Cas9-based genome editing technology [1]. The fermentation performance of the final strain (YE9) was tested under various substrate conditions, and the fermentation parameters were calculated. The dataset can be used for designing bioprocesses for pectin-rich biomass.

**Table:** Specifications Table

Subject	Applied Microbiology and Biotechnology
Specific subject area	Yeast metabolic engineering
Type of data	Tables and Figures
How data were acquired	The fermentation data were obtained by HPLC (Agilent Technologies 1260 series).
Data format	Raw and Analysed
Parameters for data collection	Fermentation conditions at 30^o^C and 130 rpm.
Description of data collection	Time series analysis of fermentation samples.
Data source location	Institution: Kyungpook National University City/Town/Region: Daegu Country: Korea
Data accessibility	With the article
Related research article	Author’s name: Deokyeol Jeong, Suji Ye, Heeyoung Park, and Soo Rin Kim Title: Simultaneous fermentation of galacturonic acid and five-carbon sugars by engineered *Saccharomyces cerevisiae* Journal: Bioresource Technology https://doi.org/10.1016/j.biortech.2019.122259

**Table:** 

**Value of the Data** • The dataset contains the construction strategy and fermentation data for the engineered strain simultaneously fermenting representative three carbon sources (xylose, arabinose, galacturonic acid) in pectin-rich biomass. • The fermentation data of the YE9 strain expressing the three pathways can be useful for process design utilizing pectin-rich biomass consisting mainly of galacturonic acid and arabinose. • Based on the fermentation data of the YE9 strain, feasible options for strain engineering can be broadened for industrial bioprocesses.

## 1 Data

**Fig. 1:**
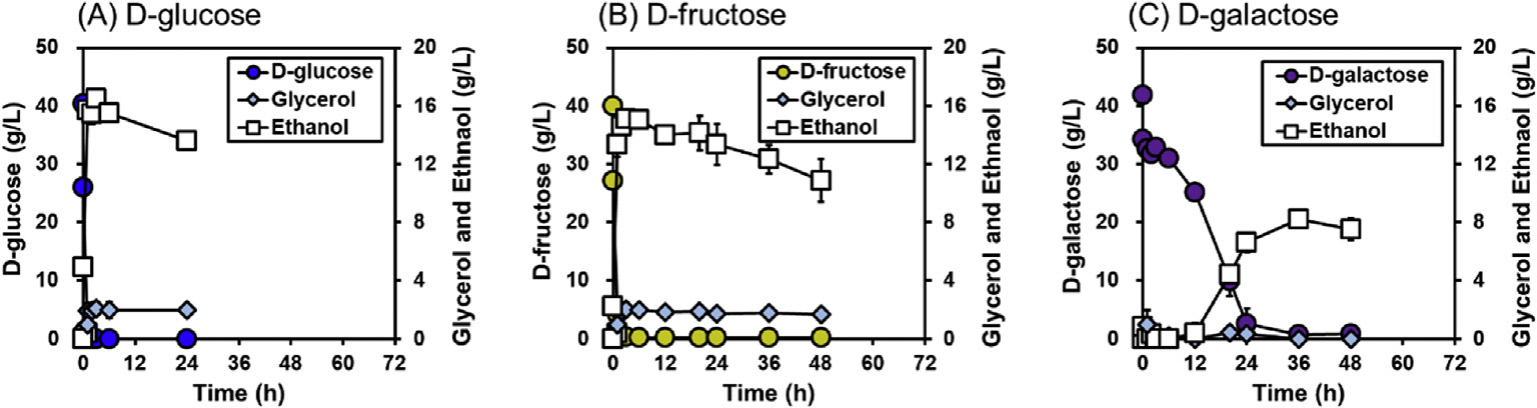
Fermentation profiles of the YE9 strain in a complex medium containing (A) 40 g/L d-glucose, (B) 40 g/L d-fructose, and (C) 40 g/L d-galactose as the sole carbon sources. Fermentations were performed under oxygen-limited conditions (130 rpm) with a starting cell density of 25 g/L. All experiments were performed in biological triplicate, and the error bars indicate the standard deviations.

**Fig. 2:**
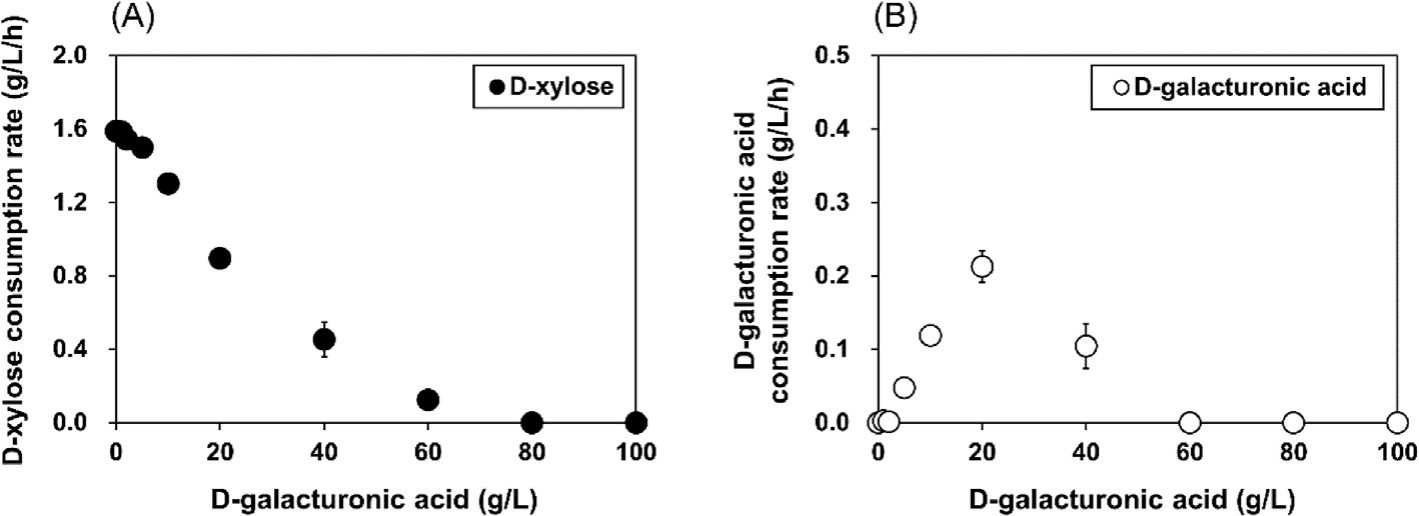
Effect of d-galacturonic acid on the rate of d-xylose consumption in the YE9 strain. Consumption rate of d-xylose (A) and d-galacturonic acid (B) was evaluated under 40 g/L D-xylose and different d-galacturonic acid concentrations (0–100 g/L). All experiments were performed in biological triplicate, and error bars indicate standard deviations and were not visible when smaller than the symbol size.

This dataset contains 1) the construction of engineered *Saccharomyces cerevisiae* strain (YE9) capable of fermenting galacturonic acid, arabionse, and xylose, and 2) its fermentation data with different carbon sources (galacturonic acid, arabinose, xylose, galactose, glucose, and fructose) and their mixtures, all of which present in pectin-rich biomass. In Fig. 1, the fermentation patterns of the YE9 strain with natively fermentable sugars (glucose, fructose, and galactose) as a sole carbon source are presented. In Table 1, the fermentation profiles of the YE9 strain with xylose, arabinose, and galacturonic acid in comparison to its wild type strain (D452-2). In Fig. 2, the YE9 strain was tested for xylose and galacturonic acid consumption rates in a mixture of 40 g/L xylose and various galactornic acid concentrations. In Table 2, the fermentation parameters of the YE9 strain with a mixture of galacturonic acid and co-substrates.

**Table 1:** Fermentation profiles of the native *S. cerevisiae* strain (D452-2) and engineered strain (YE9) expressing heterologous pathways for metabolizing d-xylose, l-arabinose, and d-galacturonic acid (galUA).

Strain			Substrate	Substrate consumed (g/L)	Substrate consumption rate (g/L/h)	Products (g/L)		Parametersb)		
						Glycerol	Ethanol	*Y*_Glycerol_	*Y*_Ethanol_	*P*_Ethanol_∗
D452-2			D-xylosea)	5.9 ± 0.2	0.19 ± 0.01	0.3 ± 0.0	*n. d.*	0.07 ± 0.02	*n. d.*	*n. d.*
			l-arabinose	1.3 ± 0.6	0.08 ± 0.03	*n. d.*	*n. d.*	*n. d.*	*n. d.*	*n. d.*
			galUA	< 0.0	< 0.00	*n. d.*	*n. d.*	*n. d.*	*n. d.*	*n. d.*
YE9			d-xylose	33.7 ± 0.5	1.41 ± 0.02	0.6 ± 0.1	11.3 ± 0.1	0.02 ± 0.00	0.34 ± 0.01	0.05 ± 0.00
			l-arabinose	30.2 ± 0.1	0.63 ± 0.07	*n. d.*	1.9 ± 0.1	*n. d.*	0.07 ± 0.00	<0.00
			galUA	6.7 ± 0.7	0.27 ± 0.01	0.3 ± 0.1	0.3 ± 0.0	0.04 ± 0.01	0.08 ± 0.02	< 0.00

a) Fermentations were performed in a complex medium containing 40 g/L d-xylose, 40 g/L l-arabinose, or 20 g/L d-galacturonic acid under oxygen-limited conditions (130 rpm) with a starting cell density of 25 g/L. Substrate consumption rate was calculated for 24 h and the others were calculated for 72 h.

b) *Y*_Glycerol_, glycerol yield (g glycerol/g substrate); *Y*_Ethanol_, ethanol yield (g ethanol/g substrate); *P*_Ethanol_∗, specific ethanol productivity (g ethanol/g cell/h); *n. d*., not detected.

**Table 2:** Fermentation profiles of mixed culture by engineered *S. cerevisiae* YE9 strain expressing heterologous pathways metabolizing d-xylose, l-arabinose, and d-galacturonic acid (galUA).

Mediuma)	Substrate consumed (g/L)		galUA consumption rate (g/L/h)	Products (g/L)		Parametersb)			
	galUA	Sugars		Glycerol	Ethanol	*Y*_Glycerol_	*Y*_Ethanol_	*P*_Glycerol_∗	*P*_Ethanol_∗
galUA	6.7 ± 0.7	–	0.27 ± 0.01	0.3 ± 0.1	0.3 ± 0.0	0.04 ± 0.01	0.08 ± 0.02	< 0.00	< 0.00
galUA + Glucose	3.3 ± 0.2	36.7 ± 0.1	0.14 ± 0.01	2.4 ± 0.3	16.9 ± 0.2	0.06 ± 0.01	0.40 ± 0.01	0.06 ± 0.00	0.66 ± 0.01
galUA + Fructose	4.5 ± 0.3	36.1 ± 0.8	0.18 ± 0.02	2.9 ± 0.1	16.9 ± 0.5	0.07 ± 0.00	0.36 ± 0.01	< 0.00	0.65 ± 0.03
galUA + Galactose	4.6 ± 1.2	25.4 ± 7.3	0.17 ± 0.03	1.6 ± 0.7	2.4 ± 1.1	0.04 ± 0.01	0.05 ± 0.02	< 0.00	< 0.00
galUA+ Xylose	13.1 ± 0.4	33.3 ± 0.5	0.49 ± 0.02	4.5 ± 0.1	12.8 ± 0.3	0.08 ± 0.00	0.23 ± 0.01	0.01 ± 0.00	0.04 ± 0.00
galUA+ Arabinose	11.9 ± 0.7	28.4 ± 0.1	0.32 ± 0.03	4.2 ± 0.2	4.1 ± 0.5	0.11 ± 0.01	0.11 ± 0.02	< 0.00	< 0.00
galUA +Xylose (X) +Arabinose (A)	15.3 ± 0.6	33.7 ± 0.1 (X) 25.9 ± 4.4 (A)	0.49 ± 0.04	5.3 ± 0.6	16.5 ± 1.2	0.07 ± 0.00	0.22 ± 0.01	< 0.00	0.02 ± 0.00

a) Fermentations were performed in a complex medium containing 20 g/L d-galacturonic acid (galUA) and 40 g/L sugar (d-glucose, d-fructose, d-galactose, d-xylose, l-arabinose, and mixture of d-xylose and l-arabinose) under oxygen-limited conditions (130 rpm) with a starting cell density of 25 g/L. d-galacturonic acid consumption rate was calculated for 24 h and the others were calculated for 72 h.

b) *Y*_Glycerol_, glycerol yield (g glycerol/g substrates); *Y*_Ethanol_, ethanol yield (g ethanol/g substrates); *P*_Glycerol_∗, specific glycerol productivity (g glycerol/g cell/h); *P*_Ethanol_∗, specific ethanol productivity (g ethanol/g cell/h).

## 2 Experimental design, materials, and methods

### 2.1 Strain construction by Cas9-based genome editing

**Fig.3:**
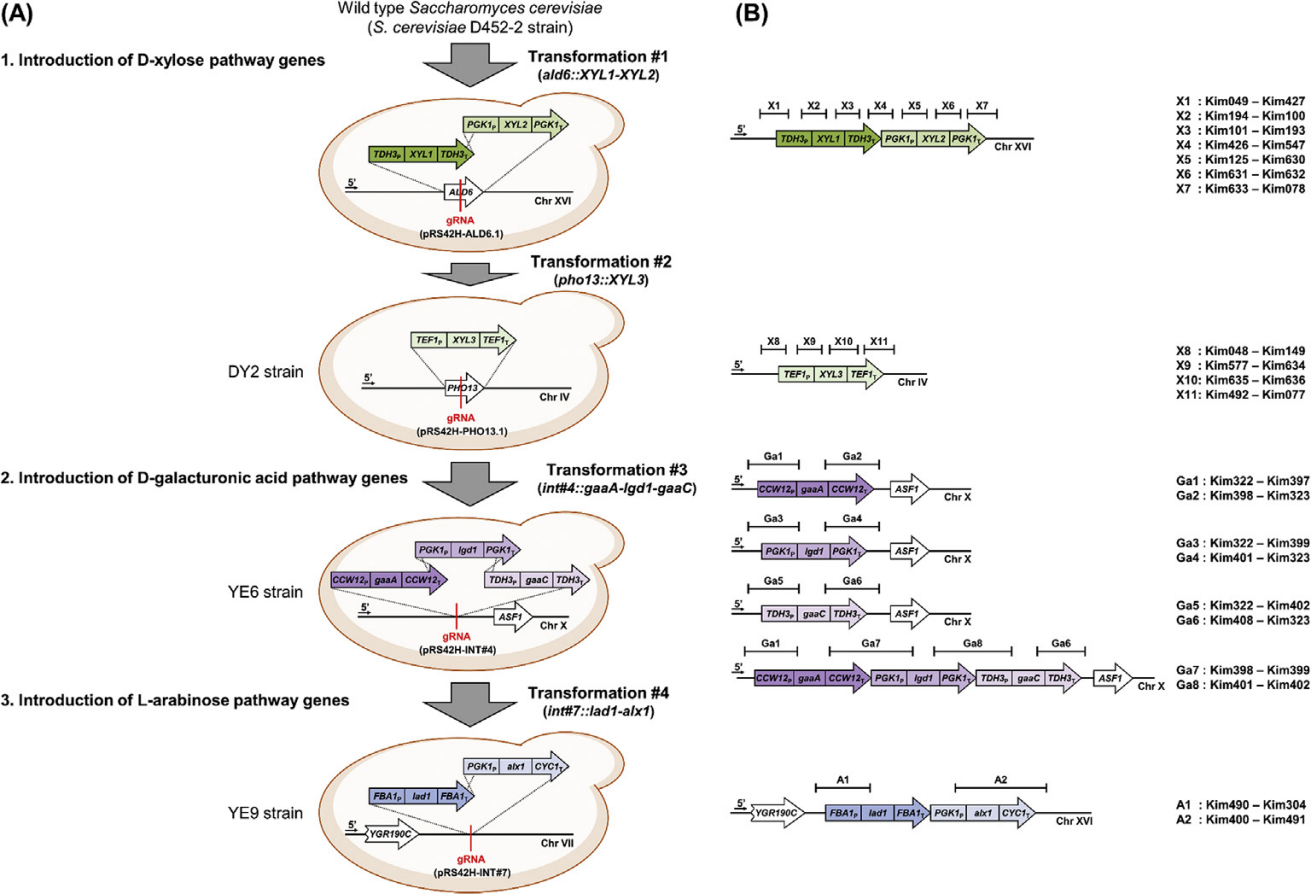
Construction of engineered *S. cerevisiae* YE9 strains expressing heterologous d-xylose, d-galacturonic acid, and l-arabinose pathways. (A) Strain construction using Cas9-based *in vivo* assembly and genome integration strategy. (B) Confirmation primers for correct assembly and integration by yeast colony PCR. The primer sequences are listed in Table S5.

To construct the YE9 strain, four consecutive transformations were performed as summarized in Fig. 3 using strains listed in Table 3. Briefly, the strain construction includes three parts: 1) guide RNA (gRNA) plasmid construction, 2) donor DNA preparation, and 3) yeast transformation.

1) Guide RNA (gRNA) plasmid construction

**Table 3:** *Saccharomyces cerevisiae* strains used for the construction of YE9.

Strains	Description/relevant genotype^a^	Ref.
D452-2	Wild type; *Matα leu2 his3 ura3*	[7]
DY02	Expressing the heterologous d-xylose pathway; D452-2 *ald6::TDH3*_P_*-XYL1-TDH3*_T_*-PGK1*_P_*-XYL2-PGK1*_T_*pho13::TEF1*_P_*-XYL3-TEF1*_T_	
YE3	DY02 *int#4::CCW12*_P_*-gaaA-CCW12*_T_	
YE4	DY02 *int#4::PGK1*_P_*-lgd1-PGK1*_T_	
YE5	DY02 *int#4::TDH3*_P_*-gaaC-TDH3*_T_	
YE6	Expressing the heterologous D-xylose and d-galacturonic acid pathway; DY02 *int#4::CCW12*_P_*-gaaA-CCW12*_T_*-PGK1*_P_*-lgd1-PGK1*_T_*-TDH3*_P_*-gaaC-TDH3*_T_	
YE6 *YPR1*	YE6 *CCW12*_P_*-YPR1*	
YE6 *gaaD*	YE6 *int#6::CCW12*_P_*-gaaD-CCW12*_T_	
YE01	Expressing the heterologous d-xylose, and l-arabinose pathway; D452-2 *ald6::TDH3*_P_*-XYL1-TDH3*_T_*-PGK1*_P_*-XYL2-PGK1*_T_*int#1::TEF1*_P_*-XYL3-TEF1*_T_*sor1::FBA1*_P_*-LAD1-FBA1*_T_*-PGK1*_P_*-ALX1-CYC1*_T_	[8]
YE9	Expressing the heterologous d-xylose, l-arabinose, and d-galacturonic acid pathway;YE6 *int#7::FBA1*_P_*-lad1-FBA1*_T_-*PGK1*_P_*-alx1-CYC1*_T_	

a *XYL1*, *XYL2*, and *XYL3* are derived from *Pichia stipitis*; *gaaA*, *gaaC*, and *gaaD* are derived from *Aspergillus niger*; *lgd1* and *lad1* are derived from *Trichoderma reesei*; *alx1* is derived from *Ambrosiozyma monospora*.

gRNA sequences are designed to be target cut site-specific and 20-bp long, as listed in Table 4. The plasmids expressing each gRNA sequence were constructed by the fast cloning method [2], which is a PCR-based protocol for plasmid mutagenesis. To construct the pRS42H-ALD6.1 plasmid, for example, the pRS42H-GND1.1 plasmid (a template plasmid) [3] was amplified with the primers Kim044/Kim045 (Table 5). The PCR products were treated with *Dpn*I and used to transform *E. coli* TOP10 (Invitrogen, Carlsbad, CA, USA). The transformants were selected on an LBA (5 g/L yeast extract, 10 g/L tryptone, 10 g/L NaCl, and 100 μg/mL ampicillin) agar plate. The gRNA sequence of the resulting plasmid was confirmed by Sanger sequencing using a universal primer for the T3 promoter. All other gRNA plasmids were constructed using the same procedure but different primers, as listed in Table 5.

2) Donor DNA preparation

**Table 4:** Guide RNA (gRNA) plasmids.

gRNA	Target cut site	gRNA and PAM sequences (5’-)	Plasmid name
ALD6.1	*ALD6*	GTCAAGATCACACTTCCAAA TGG	pRS42H-ALD6.1
PHO13.1	*PHO13*	TCCCTTATCTATTAACTTTC CGG	pRS42H-PHO13.1
YPR1.1	*YPR1*	CATGGTAGATTATTATCTGT GGG	pRS42H-YPR1.1
INT#4	Intergenic region upstream *ASF1*	CTCTCGAAGTGGTCACGTGC GGG	pRS42H-INT#4
INT#6	Intergenic region upstream *ATG33*	TTGTCACAGTGTCACATCAG CGG	pRS42H-INT#6
INT#7	Intergenic region downstream *YGR190C*	GATACTTATCATTAAGAAAA TGG	pRS42H-INT#7

**Table 5:** Primers used for construction of guide RNA plasmids.

Plasmid name	Primers	Sequences (5’-)
pRS42H-ALD6.1	Kim044	AAGATCACACTTCCAAAGTTTTAGAGCTAGAAATAGCAAG
	Kim045	TTGGAAGTGTGATCTTGACGATCATTTATCTTTCACTGCG
pRS42H-PHO13.1	Kim624	CTTATCTATTAACTTTCGTTTTAGAGCTAGAAATAGCAAG
	Kim625	AAAGTTAATAGATAAGGGAGATCATTTATCTTTCACTGCG
pRS42H-YPR1.1	Kim535	GGTAGATTATTATCTGTGTTTTAGAGCTAGAAATAGCAAG
	Kim536	CAGATAATAATCTACCATGGATCATTTATCTTTCACTGCG
pRS42H-INT#4	Kim310	TCGAAGTGGTCACGTGCGTTTTAGAGCTAGAAATAGCAAG
	Kim311	CACGTGACCACTTCGAGAGGATCATTTATCTTTCACTGCG
pRS42H-INT#6	Kim314	TCACAGTGTCACATCAGGTTTTAGAGCTAGAAATAGCAAG
	Kim315	TGATGTGACACTGTGACAAGATCATTTATCTTTCACTGCG
pRS42H-INT#7	Kim486	AGGAATTATGTTCGCCCGTTTTAGAGCTAGAAATAGCAAG
	Kim487	GGCGAACATAATTCCTTACGATCATTTATCTTTCACTGCG

Donor DNA fragments were prepared by PCR using the primers listed in Table 6. Each of the fragments was flanked by 40–50 bp to allow *in vivo* assembly and genome integration through homologous recombination. Each assembly was an expression cassette of a heterologous gene as described in Fig. 3A. The donor DNAs for the xylose expression cassettes were designed to achieve complete removal of a target gene when genome integrated. On the other hand, the expression cassettes of the arabinose pathway and galacturonic acid pathway were integrated into an intergenic region without interfering neighboring genes.

3) Yeast transformation

**Table 6:** Primers used for construction of donor DNA fragments.

Template genomic DNA^a^	Donor DNA fragments	Primers	Sequences (5’-)
***XYL1* and *XYL2* expression cassettes for deleting *ALD6* (*ald6::TDH3***_**P**_***-XYL1-TDH3***_**T**_***-PGK1***_**P**_***-XYL2-PGK1***_**T**_**)**			
*S. cerevisiae*	*TDH3*_P_	Kim626	TAACATACACAAACACATACTATCAGAATACACTATTTTCGAGGACCTTGTC
		SOO384	TCAACTTAATAGAAGGCATTTTTAGATCTCCTAGGTTTGTTTGTTTATGTGTGTTTAT TC
*P. stipitis*	*XYL1*	SOO385	ATAAACACACATAAACAAACAAACCTAGGAGATCTAAAAATGCCTTCTATTAAGTTGA AC
		SOO386	AAT GCAAGATTTAAAGTAAATTCACTGTTAACGCATGCTTAGACGAAGATAGGAATCTTG
*S. cerevisiae*	*TDH3*_T_	SOO387	GGA CAAGATTCCTATCTTCGTCTAAGCATGCGTTAACAGTGAATTTACTTTAAATCTTGC
		SOO388	ATTCTTTGAAGGTACTT CTTCGAAAAATTCGCGTCTGCTAGCTCCTGGCGGAAAAAATTC
*S. cerevisiae*	*PGK1*_P_	SOO389	TTTTAAAGTTTACAAAT GAATTTTTTCCGCCAGGAGCTAGCAGACGCGAATTTTTCGAAG
		SOO390	CACCAA GGAAGGGTTAGCAGTCATTTTTTCTAGATGTTTTATATTTGTTGTAAAAAGTAG
*P. stipitis*	*XYL2*	SOO391	AATTAT CTACTTTTTACAACAAATATAAAACATCTAGAAAAAATGACTGCTAACCCTTCC
		SOO392	AAAAAATTGAT CTATCGATTTCAATTCAATTCAATACTAGTTTACTCAGGGCCGTCAATG
*S. cerevisiae*	*PGK1*_T_	SOO393	GTCAAGTGTCT CATTGACGGCCCTGAGTAAACTAGTATTGAATTGAATTGAAATCGATAG
		Kim627	GTATATGACGGAAAGAAATGCAGGTTGGTACA AAATAATATCCTTCTCGAAAG
***XYL3* expression cassette for deleting *PHO13* (*pho13::TDH3***_**P**_***-XYL1-TDH3***_**T**_***-PGK1***_**P**_***-XYL2-PGK1***_**T**_**)**			
*S. cerevisiae*	*TDH3*_P_	Kim628	ATGTGACATCTTTACTATTCTCCAGCACGTTT CTTCATCGGTATCTTCGC
		SOO374	AA TGGGGTAGTGGTCATTTTTAAGCTTGAATTCTTTGTAATTAAAACTTAGATTAGATTG
*P. stipitis*	*XYL3*	SOO375	AT CTAATCTAAGTTTTAATTACAAAGAATTCAAGCTTAAAAATGACCACTACCCCATTTG
		SOO376	GCAACTA GAAAAGTCTTATCAATCTCCGTCGACATCGATTTAGTGTTTCAATTCACTTTC
*S. cerevisiae*	*TDH3*_T_	SOO377	CAAGATG GAAAGTGAATTGAAACACTAAATCGATGTCGACGGAGATTGATAAGACTTTTC
		Kim629	CTATAACTCATTATTGGTTAAGGTGTAGATG AAGTTGGGTAACGCCAGG
***gaaA* expression cassette (*int#4::CCW12***_**P**_***-gaaA-CCW12***_**T**_**)**			
*S. cerevisiae*	*CCW12*_P_	Kim379	TTCCTCGGGCAGAGAAACTCGCAGGCAACTTG CACGCAAAAGAAAACCTT
		Kim380	TCAACA CAGCTGGGGGAGCCATTTTTTATTGATATAGTGTTTAAGCGAAT
*A. niger*	*gaaA*	Kim381	TCTGTC ATTCGCTTAAACACTATATCAATAAAAAATGGCTCCCCCAGCTG
		Kim382	TAGA ATGTATAAATAATAATAAACTAAGTCTACTTCAGCTCCCACTTTCC
*S. cerevisiae*	*CCW12*_T_	Kim383	GGAT GGAAAGTGGGAGCTGAAGTAGACTTAGTTTATTATTATTTATACAT
		Kim384	TGTGAGGGCCGATTATGCAGGCCTAGA TGTTCTAGTGTGTTTATATTATC
***lgd1* expression cassette (*int#4::PGK1***_**P**_***-lgd1-PGK1***_**T**_**)**			
*S. cerevisiae*	*PGK1*_P_	Kim385	CCTCGGGCAGAGAAACTCGCAGGCAACTTG GTGAGTAAGGAAAGAGTGAG
		Kim386	GTGATGGTGACTTCAGACATTTTTTGTTTTATATTTGTTGTAAAAAGTAG
*T. reesei*	*lgd1*	Kim387	CTACTTTTTACAACAAATATAAAACAAAAAATGTCTGAAGTCACCATCAC
		Kim388	ATTGATCTAT CGATTTCAATTCAATTCAATTCAGATCTTCTCTCCGTTCA
*S. cerevisiae*	*PGK1*_T_	Kim389	CTGCCCATCT TGAACGGAGAGAAGATCTGAATTGAATTGAATTGAAATCG
		Kim390	CTCTGTGAGGGCCGATTATGCAGGCCTAGA AAATAATATCCTTCTCGAAA
***gaaC* expression cassette (*int#4::TDH3***_**P**_***-gaaC-TDH3***_**T**_**)**			
*S. cerevisiae*	*TDH3*_P_	Kim391	CTCGGGCAGAGAAACTCGCAGGCAACTTG GAATAAAAAACACGCTTTTTC
		Kim392	GACTCCGGGGCG GAGCGGGGTAAAAGGCATTTTTTTTGTTTGTTTATGTGTGTT
*A. niger*	*gaaC*	Kim393	TTCGAATA AACACACATAAACAAACAAAAAAAATGCCTTTTACCCCGCTC
		Kim394	ATTTAAAT GCAAGATTTAAAGTAAATTCACCTAAGCAATATCCGGCAACG
*S. cerevisiae*	*TDH3*_T_	Kim395	TGAGAAGT CGTTGCCGGATATTGCTTAGGTGAATTTACTTTAAATCTTGC
		Kim396	CCTCTGTGAGGGCCGATTATGCAGGCCTAGA ATCCTGGCGGAAAAAATTC
***gaaA*, *lgd1*, and *gaaC* expression cassettes (*int#4::CCW12***_**P**_***-gaaA-CCW12***_**T**_***-PGK1***_**P**_***-lgd1-PGK1***_**T**_***-TDH3***_**P**_***-gaaC-TDH3***_**T**_**)**			
*S. cerevisiae* YE3	*CCW12*_P_*-gaaA-CCW12*_T_	Kim410	TCTTTAGGTTAATTGTCGCTGTTATTGTCTA GATTTTTTCTCGGAGATGG
		Kim411	TAGTTC CTCACTCTTTCCTTACTCACTGTTCTAGTGTGTTTATATTATCC
*S. cerevisiae* YE4	*PGK1*_P_*-lgd1-PGK1*_T_	Kim412	AGCCAA GGATAATATAAACACACTAGAACA GTGAGTAAGGAAAGAGTGAG
		Kim413	AAACTCGAA CTGAAAAAGCGTGTTTTTTATTCCCGATTATGCAGGCCTAG
*S. cerevisiae* YE5	*TDH3*_P_*-gaaC-TDH3*_T_	Kim414	TATTATTTT CTAGGCCTGCATAATCGGGAATAAAAAACACGCTTTTTCAG
		Kim415	CTACTCTCTTCCTAGTCGCCCGGTTGTT GAAAGTTTAATTGTGGGTTTTC
***lad1 and alx1* expression cassettes (*int#7::FBA1***_**P**_***-lad1-FBA1***_**T**_**-*PGK1***_**P**_***-alx1-CYC1***_**T**_**)**			
*S. cerevisiae* YE01	*FBA1*_P_*-lad1-FBA1*_T_-*PGK1*_P_*-alx1-CYC1*_T_	Kim553	CTTACACTTGTGTAATGACAAATGTTTTT TGAACAACAATACCAGCCTTC
		Kim554	TGTTTCACGTTATCAAGATTATGTCATCTATT GGCCGCAAATTAAAGCCT
**Overexpression of *YPR1* (*CCW12***_**P**_***-YPR1*)**			
*S. cerevisiae*	*CCW12*_P_	Kim537	GTAACTTTGCAATATAATCAGGTCGCAAATAT CACGCAAAAGAAAACCTT
		Kim538	GAAGAATTCTTTAACGTAGCAGGCAT TATTGATATAGTGTTTAAGCGAAT
***gaaD* expression cassette (*int#6::CCW12***_**P**_***-gaaD-CCW12***_**T**_**)**			
*S. cerevisiae*	*CCW12*_P_	Kim541	CGGAGGAGACCGCTATAACCGGTTTGAATTTA CACGCAAAAGAAAACCTT
		Kim542	TA ACCTTCTTTCCGAGAGACATTTTTTATTGATATAGTGTTTAAGCGAAT
*A. niger*	*gaaD*	Kim543	TC ATTCGCTTAAACACTATATCAATAAAAAATGTCTCTCGGAAAGAAGGT
		Kim544	GT ATAAATAATAATAAACTAAGTTTATTAAACAATCACCTTATGACCAGC
*S. cerevisiae*	*CCW12*_T_	Kim545	TG GTCATAAGGTGATTGTTTAATAAACTTAGTTTATTATTATTTATACAT
		Kim546	CTTGCTTGCTGTCAAACTTCTGAGTTG TGTTCTAGTGTGTTTATATTATC

The flanking region is underlined.

a *Saccharomyces cerevisiae* D452-2; *Pichia stipitis* CBS 6054; *Aspergillus niger* CBS 120.49; *Trichoderma reesei* ATCC 5676.

For yeast transformation, a gRNA plasmid (4 μg) and donor DNA fragments (4 μg each) were used to transform a designated strain harboring pRS41N-Cas9 [3]. The resulting transformants were selected on a YPD agar plate supplemented with 100 μg/mL nourseothricin sulfate (Gold Biotechnology, St. Louis, MO, USA) and 300 μg/mL hygromycin B (Invitrogen, Carlsbad, CA, USA). Selected transformants were serially sub-cultured in YPD medium supplemented with 100 μg/mL nourseothricin sulfate to only remove the existing gRNA plasmids. Correct assembly and integration was then confirmed by yeast colony PCR with the primers listed in Table 7. Through four consecutive transformations, as described in Fig. 3, the YE9 strain was finally constructed.

**Table 7:** Primers used for confirmation of correct assembly and integration.

Primers	Sequences (5’-)	Primers	Sequences (5’-)
Introduction of d-xylose pathway		Introduction of d-galacturonic acid pathway	
Kim049	GGAACGGTGAGTGCAACG	Kim322	GCGCATCTATTTGCCGTC
Kim427	AAACTGTTCACCCAGACACC	Kim397	GCTGGGGGAGCCATTTTTTATTG
Kim194	AGCGCAACTACAGAGAACAGG	Kim398	GTGGGAGCTGAAGTAGACTTAG
Kim100	CGGCACCGTCGAACAATCTG	Kim323	TCACGACACACCTCACTG
Kim101	CCGCTTACTCTTCGTTCGGTCC	Kim399	CCTGTGATGGTGACTTCAGAC
Kim193	CTCAGCATCCACAATGTATCAG	Kim401	GAACGGAGAGAAGATCTGAATTG
Kim426	GCGCTATTGCATTGTTCTTGTC	Kim400	ACAGCCTGTTCTCACACAC
Kim547	AGGTATGCGATAGTTCCTCAC	Kim402	GCGGGGTAAAAGGCATTTTTTTTG
Kim125	TGCAGCTTCCAATTTCGTCAC	Kim408	GCCGGATATTGCTTAGGTG
Kim630	GAGGTGACACCCTTACCAAC		
Kim631	CTGCTACTCACACCTTCAACTC	**Introduction of l-arabinose pathway**	
Kim632	CGCTGAACCCGAACATAGAAATATC	Kim490	GGCACTAGGAGCATTTGTCG
Kim633	TCGATATTTCTATGTTCGGGTTCAG	Kim304	GCTTCGCTAATCCAGAGGTC
Kim078	GATTGGAATTGGTTCGCAGTG	Kim400	ACAGCCTGTTCTCACACAC
Kim048	GAGGAAGACGTTGAAGGTGG	Kim491	GTCCCTTAGGGTGCGTATAATG
Kim149	TTTGAAGTGGTACGGCGATG		
Kim577	CACCCAAGCACAGCATAC	**Overexpression of *YPR1***	
Kim634	TGGCTCGATAACGAAGATTCAG	Kim539	CAATTCCGTGAAACCCTTTTCTT
Kim635	GTCTTGTAGATTGAGAACTGGTCC	Kim540	CTGCCAACTTCTTCTTCATTCAA
Kim636	TCTATGAGGCAAGTAAGAGGCAC		
Kim492	AACAGGCGACAGTCCAAATG	**Introduction of *gaaD* gene cassette**	
Kim077	TTGGAGTTCAAACTGGCGAG	Kim326	GGTTCTGACTCCTACTGAGC
		Kim093	GCAAAGATAGCGGCGTAGGTG
		Kim549	GCATCCTTTGCCTCCGTTC
		Kim327	AGCATCGAGTACGGCAGTTC

### 2.2 Fermentation

For fermentation of the YE9 strain, one colony was pre-cultured in YP medium (10 g/L yeast extract and 20 g/L peptone) supplemented with 20 g/L of glucose for 36 h at 30^o^C and 250 rpm. Cells were centrifuged, washed twice, and re-suspended in YP medium supplemented with desired carbon sources. The initial cell density of fermentation was 25 g/L dry weight, which corresponds to approximately 125 g/L wet weight, and this conversion factor was obtained from a prior study [4]. In the industrial bioethanol processes, >90% cells are recycled in repeated batch-type fermentation; therefore, very high cell density of up to 170 g/L wet weight [5] is often achieved. The concentrations of the carbon sources were selected to reflect the typical chemical composition of pectin-rich biomass (Table 8).

**Table 8:** Chemical composition of pectin-rich biomass.

Source	Arabinose	Galacturonic acid	Ratio	Reference
Orange peel hydrolysate (g/L, ∼ 10% solid loading)	32.6	13.2	2.47	[9]
Sugar beet pulp hydrolysate (g/100 g dry matter)	22.5	22.5	1.00	[10]

### 2.3 HPLC analysis

Quantitation of glucose, fructose, galactose, xylose, arabinose, galacturonic acid, glycerol, and ethanol was performed by high-performance liquid chromatography (HPLC; Agilent Technologies, 1260 series, USA) device equipped with a RI detector and a Rezex-ROA Organic Acid H+ (8%) (150 mm × 4.6 mm) column (Phenomenex Inc., Torrance, CA, USA). The column was eluted with 0.005 N H_2_SO_4_ at 0.6 mL/min and 50^o^C [1,6].
